# The “anti-vax” movement: a quantitative report on vaccine beliefs and knowledge across social media

**DOI:** 10.1186/s12889-021-12114-8

**Published:** 2021-11-17

**Authors:** Staci L Benoit, Rachel F. Mauldin

**Affiliations:** Saint James School of Medicine, Park Ridge, Illinois USA

**Keywords:** Social media, Vaccine, Anti-vax, Vaccine denier, Facebook, Twitter, Instagram, Social network

## Abstract

**Background:**

Social media use has become a mainstay of communication and with that comes the exchange of factual and non-factual information. Social media has given many people the opportunity to speak their opinions without repercussions and create coalitionS of like-minded people. This also has led to the development of a community know as anti-vaxxers or vaccine deniers. This research explores the extent to which vaccine knowledge has reached on social media.

**Methods:**

This cross sectional research explored the relationship between the spread of information regarding vaccines in relation to social media use. A sample of 2515 people over the age of 18 around the world completed the survey via a link distributed on Twitter, Facebook and Instagram. A series of questions on vaccine knowledge and beliefs were compounded to create an individual’s “knowledge score” and a “belief score”. Knowledge scores were ranked from low knowledge to high knowledge with increasing scores. Belief scores were ranked from belief in myths to disbelief in myths with higher scores. This score was then analysed, using a Welch test and post hoc testing when applicable, across demographics and questions relating to social media use.

**Results:**

Significant relations were found in both the knowledge and belief categories, many of which were similar findings between the two. North Americans had significantly lower knowledge and belief scores compared to all other continents. While the majority of people primarily use Facebook, Twitter users were significantly more knowledgeable. It was also found that higher education was correlated with higher knowledge and belief scores.

**Conclusions:**

Overall, these correlations are important in determining ways to intervene into the anti-vax movement through the use of social media. Cross demographics were not analysed in this study but could be in future studies. To better understand the social media exposures related to vaccine information a follow up structured interview research study would be beneficial. Note that due to the cross sectional nature of this study, causal relationships could not be made.

**Supplementary Information:**

The online version contains supplementary material available at 10.1186/s12889-021-12114-8.

## Background

Fear of vaccines dates as far back as vaccines themselves as evident by Edmund Massey's [29] Sermon titled “A sermon against the dangerous and sinful practice of inoculation.” This appears to be the first objection to any forms of inoculation to prevent disease with Massey stating “Let us not sinfully endeavour to alter the Course of Nature” [29]. Next in notable vaccine objections was when the smallpox vaccine was introduced, “many skeptics […] found it counterintuitive that deliberately infecting a person with a disease” [38]. This is when the world began to see a group of people who not only refused vaccination but made an effort to inform others of the “dangers” through propaganda. However this propaganda largely consisted of arguments of infringement of rights and anti-socialism [15].

Since the infamous 1998 paper by Andrew Wakefield, this was later retracted because it incorrectly related the MMR vaccine to autism, a group of people known as vaccine deniers or more commonly known as anti-vaxxers have been exponentially growing. A vaccine denier or anti-vaxxer will be defined in this study as someone who believes vaccines do not work, are not safe or refuse vaccines for themselves and their children if applicable. Claims about vaccine safety, efficacy, and adverse effects have been evolving and have now spread to almost every vaccine available. Surveys from the American Academy of Pediatrics found that the rate of parents who refused one or more recommended vaccines increased from 9.1% in 2006 to a staggering 16.7% in 2013 [30]. The problem being faced today is the wealth of information that is not only accessible but easily spread across social media platforms regardless of veracity. It is clear that the internet is now patching a significant time in health literacy and decision making. A survey by Fox [18] found "(72%) [Of] adult internet users say they have searched online for information about a range of health issues[...] (26%) adult internet users say they have read or watched someone else’s health experience about health or medical issues in the past 12 months. And 16% of adult internet users in the U.S. have gone online in the past 12 months to find others who share the same health concerns. There are an estimated 58 milion followers on anti-vaccination pages across socialmedia ]^1^]. This study uses Social media propagation, to reach the study population.

The purpose of this study is to evaluate the current knowledge and beliefs about vaccines in people who use social media and the differences between scores and demographics. Past research has found that the strongest influence for positive vaccine views is having factual knowledge [11]. This study hopes to determine how much factual information is known and what differentiates social media users who have adequate and inadequate knowledge. This cross sectional study hypothesizes that there are correlations between each of the individual demographic questions and the respondent’s knowledge of vaccines. Knowing what differentiates people who have adequate and inadequate knowledge can be beneficial for determining how to reach people in future vaccine campaigns. This information could also be used in an attempt to better educate or correct misinformation by means of social media in those who classify under this study as having insufficient knowledge. Additionally this research will investigate the individual’s beliefs about vaccinations in people who use social media, whether positive or negative. Uncovering individual characteristics in the respondents with their different beliefs can be helpful for deciding how to correctly reach each person group in the future in regards to vaccinations. We can use this information for educational purposes through social media to help those who were found to have negative or lower beliefs toward vaccinations.

Knowledge about vaccines, both true and false, can be easily accessible but also easily confused. The internet has become a huge influence on vaccine knowledge [26] and the emergence of social media has created a vast community that allows multi-person discussion to happen instantaneously and with little supervision [9]. “Anti-vaccination activists use [social media] to disseminate messages, facts and beliefs that oppose some or all recommended vaccinations” [6]. Brunson [8] found that the most significant factor influencing parents online is the percent of parents within a parents online network that are nonconforming. Because of these factors refusal to vaccinate is becoming a concern of public safety.

Regardless of vaccination rates, legitimate information about vaccine safety is “not reaching parents in an effective or convincing manner” [19]. This study seeks to assess whether factual knowledge is reaching social media users. This is because it has been found that the strongest predictor of positive attitudes towards vaccines is better knowledge [11]. In a study of Serbian University students it was found that 47.9% of participants thought that giving multiple vaccines at one time overloads the immune system [11]. Further research will determine if views such as the one above are perceived across all social media users and not just students.

Investigations of the dissemination of vaccine knowledge across social media have been substantially less studied until recent years, especially with the development of the COVID-19 vaccine. In a study of vaccine attitudes in Twitter users Mitra et al. found that anti-vaccination tweets had a wider reach (seen by more people) than pro-vaccine tweets [31]. In a study of HPV vaccine tweets it was found that there was “an association between prior exposure to negative tweets about HPV vaccines and the subsequent posting of negative tweets about HPV vaccines” which allowed for the sharing of negative opinions to more susceptible people [14]. From the information aforementioned it can be predicted that the majority of information about vaccines on social media has a negative connotation. What is still unclear is the ability of the general population to distinguish between fact and fiction, and whether this information has an influence on their knowledge and eventually decisions.

To determine what questions would be used and what is to be expected by demographic results this study looked at Duggan and Brenner's [13] journal article on social media users. This study found several useful social media user characteristics. Firstly, women are statistically significantly more likely to be social media users compared to men. This study also shows that with age, there is a statistically significant progressive decline in social media use. In terms of education they found that social media use was approximately equal across all levels of education. Equity across races was also seen. This demographic data is important for determining what questions should be asked, but also what can be expected in the results.

It is important to determine what factors influence people to not vaccinate. Yaqub et al. found that “‘distrust of doctors’, ‘distrust of government sources’, and ‘distrust of pharmaceutical companies’ as reason for hesitancy” [40]. In that same study they found that very few people reported that they did not have access to adequate information. While this is important, it does not elaborate on the quality of information these people are using. A cross sectional study in Australia in 2012 found that although 92% of 452 parents reported that their children were adequately vaccinated, 52% reported concerns including but not limited to vaccine safety and source of vaccine knowledge [10]. However due to discrepancies in defining anti-vaxxers or people who are vaccine hesitant, this study will focus on what knowledge people have and not ask their vaccine practices. Rather, it will look into how far the anti-vax messages have reached and the characteristics of those participants that have or have not been swayed by these messages.

Since the collection of the data related to this research the world has faced the COVID-19 pandemic. Social media and vaccine hesitancy has become a huge topic of discussion and research relating to combating COVID-19. Several studies worldwide have indicated that older individuals, females, those with higher incomes and those with higher education levels were more likely to accept a vaccine [34]. Research in this area has exploded due to fast paced development and deployment of the COVID 19 vaccine. Globally there has been varying intention to get the COVID-19 vaccine ranging from 41 and 89% [17]. A survey conducted in the United States of America showed only 57.6% of respondents intended to be vaccinated but also that “(62%) [of respondents] believed that sociopolitical factors and pressures may lead to a rushed approval for the COVID vaccine without the assurances of safety and efficacy” [27].

## Methods

### Collection

This research was conducted by a cross sectional multiple choice study created via Survey Monkey. Survey Monkey subscription was provided by Saint James School of Medicine and was chosen based on the platforms ability to export data to IBM's SPSS. Data of both qualitative (demographic) and quantitative (Belief and Knowledge Scores as described below) nature were collected. Survey was designed to be completed in 5–10 min. It was designed with no open ended questions, no intended question bias, and no implied judgement. Survey questions were validated by pilot participation and follow up to determine if there was any ambiguity that needed to be addressed. Demographic questions were designed to be all inclusive. Belief and Knowledge questions were based off of key arguments of vaccine deniers as determined by popular social media posts. Research of key argument involved exploring multiple social media platforms and investigating posts/comments regarding vaccine denial.

### Inclusion/exclusion

Subjects were included based on completion of all parts of the survey. All demographics (country, race, gender, socioeconomic status, preferred social media platform) were included except for those under 18 years of age. Those participants under 18 were excluded because in most countries the age at which an individual can consent for medical treatment such as vaccination is 18 year old [39]. Therefore, vaccine decision making if deferred to parents and guardians over 18 years of age. Those participants who no do not consent to their information being used for research purposes (first question of the survey) were excluded from the study. Survey’s that contained any missing data/questions were excluding from analysis as scoring could not be completed. The survey was designed to not allow advancement onto next question without answering the current question. If participants clicked out of browser before completing final question their survey was invalidated and subsequently not extrapolated for analysis. Survey was available for completion from August 15, 2018 till November 1, 2018.

### Sampling

Snowball sampling of social media users was used. Snowball sampling was used to help perpetuate the survey through social media, where social media is the quality of referral. The study population was aimed at being as demographically diverse as possible among people who use social media. Snowball sampling was chosen in particular for its ability to perpetuate hard to reach communities, such as those who identify as vaccine deniers [23]. Additionally, using Facebook with snowball sampling is effective at diversifying the geographical scope and increasing the overall response rate [3].

### Recruitment

Subjects were recruited through the three largest social media platforms (Facebook, Twitter, Instagram) via a shareable web link and asked to consent before completion of the research survey. Initial survey link was posted publicly on social media platforms outlined above with information on the survey and encouragement to further share the survey once completed. A web survey was chosen due to its ease, speed, cost, and ability to obtain a geographically diverse population [20].

### Questions

All questions in the survey were only available in English language and can be found in the supplementary material, document 1 titled Survey. The first half of the survey consisted of demographics and questions pertaining to use of social media and its relation to vaccine information. The latter half of the survey had six questions relating to vaccine knowledge and six questions relating to vaccine myths. The method of scoring was designed by the authors to create a numerical scale for comparative analytics. Lack of knowledge and belief in myths is not a negative feature but more so an area of improvement and discussion. As such, the design only uses positive numerals for scoring each question. This scoring system was created by the authors of this study specifically for this research. Question content was selected by authors through observation of social media posts pertaining to vaccines (both pro vaccine and anti-vaccine content) for common misconceptions and rebuttals.

The six vaccine knowledge questions were scored on a two point scale. Questions were scored by awarding two points for the answer of belief in the vaccine statement, one point for uncertainty, and zero points for the answer of disbelief in the statement. All questions were then totaled for a score on a 12 point scale. Higher values (9–12) suggesting adequate vaccine knowledge, middle range (5–8) suggesting some vaccine knowledge but with uncertainty and lower values (0–4) suggesting inadequate vaccine knowledge. This score could then be appropriately analyzed.

The six vaccine belief questions were scored on a two point scale. Two points were given for the answer choice “disbelief in the vaccine statement”, one point for uncertainty, and zero points for the answer of belief in the statement. All questions were then totaled for a score on a 12-point scale. Similar to knowledge values, higher values were indicative of disbelief in common myths, whereas lower values indicated a belief in common myths. This score could then be appropriately analyzed.

### Analysis

All data analysis was conducted using IBM’s SPSS. Significance testing was performed using the Welch test. This test was chosen based on the negatively skewed data distribution with non-homogeneity of variances and sample sizes [16]. The Welch test has historically been shown to better control Type 1 error for these parameters compared to other tests [35]. Post hoc analysis was completed with Games Howell due to its robustness and utility in non-normal distributions [21]. A standard *P* value of 0.05 was used for statistical significance but reported up to < 0.001 which is the limit on statistical software. Raw and descriptive data is available from openICPSR.org project ID openicpsr-120,505 [5].

## Results

Of the 2517 respondents, 2417 were included in the analysis based on the inclusion/exclusion criteria. The age of participants showed 446 (18.5%) people aged 18–24, 715 (29.6%) people aged 25–34, 591 (24.5%) people aged 35–44, 394 (16.3%) people aged 45–54, 189 (7.8%) people aged 55–64 and 82 (3.4%) people over the age of 65. Females accounted for 80.1% of the respondents (*n* = 1937 people) and males accounted for 18.8% (*n* = 454 people). Respondents were predominantly North American with 70.3% (*n* = 1700) from the USA and 12.9% (*n* = 312) from other North American Countries. The remaining were divided into 7.4% (*n* = 180) Australia/Oceanic, 7.3% (*n* = 176) European, 0.9% (*n* = 22) Asian, 0.6% (*n* = 15) African, 0.5% (*n* = 11) South American, and 1 respondent from Antarctica. The education levels showed that 0.1% (*n* = 2) had no formal schooling, 0.4% (*n* = 10) completed elementary school (grade level 1–8), 21.2% (*n* = 509) completed high school (grade level 9–12/13), 22.3% (*n* = 540) completed an Associates (2 year) degree, 34.5% (*n* = 833) completed a Bachelor (4 year) degree, 13.9% (*n* = 336) completed a Master’s degree, and 7.7% (*n* = 187) completed a Professional degree (PhD, MD, DC, DO, etc.). Individuals identified themselves as Lower class socioeconomic status (SES) comprised 9.8% (*n* = 238) of the population, 82.2% (*n* = 1987) as Middle class SES, and 7.9% (*n* = 192) as Upper class SES.

Facebook is the most commonly used social media type in the population with 69.8% (*n* = 1688), followed by Twitter 15.6% (*n* = 378), Instagram 12.9% (*n* = 311), then other forms of social media 1.7% (*n* = 40). Other forms of social media were identified as Snapchat, Tumblr, Reddit, Pinterest, or using all platforms equally. Most people 47.9% (*n* = 1158) claimed to only spend 0–2 h on social media daily, followed by 40.9% (*n* = 989) using 3–4 h, 8.4% (*n* = 204) using 5–6 h, 1.5% (*n* = 36) using 7–8 h, and 1.2% (*n* = 30) using social media for over 9 h. Most respondents 92.7% (*n* = 2240) have seen posts on social media about vaccines and only 7.3% (*n* = 177) have not. These posts influence 5.4% (*n* = 130) of users to think vaccines are worse than previously thought, 13.6% (*n* = 328) to think vaccines are better than previous thought, 76.4% (*n* = 1846) claim to not have been influenced by the posts, and 4.7% (*n* = 113) had not seen any posts. Lastly, people claimed to trust doctors 89.4% (*n* = 2160) the most with their immunization related information/decisions. The remaining people trust the internet 4.1% (*n* = 100), family 2.0% (*n* = 48), peers and friends 2.3% (*n* = 55), social media 0.2% (*n* = 5) and the government 2.0% (*n* = 49) with their information and decisions.

### Knowledge

Table 1, found in the supplementary document 2 labeled “Tables”, depicts the frequency of knowledge scores in the sample population. As described in the methods, knowledge scores are based on a scale from 0 to 12 derived from 6 questions with answers ranked from 0 to 2 points. Scores toward 0 represent negatively skewed knowledge, or lack of correct information. Scores toward 12 represent positively skewed knowledge, or adequate vaccine knowledge. Scores of 6 represent uncertainness.

Analysis of all demographic questions against the respondent’s knowledge score was completed by Welch and then further analyzed by Games Howell. Explanations of why these tests were chosen can be found in Methods. When age was compared with knowledge scores a Welch statistical value of 0.763 and the significance of 0.576 (*p* > 0.05). Post hoc was not necessary.

Gender analysis showed a Welch statistic of 1.627 with a significance value of 0.204 (*p* > 0.05). Post hoc analysis was not examined because there was no significance.

Geographical Welch testing showed a statistic value of 11.552 with a significance of < 0.001(*p* < 0.05). Since this value is statically significant post hoc analysis was examined. North Americans (USA) has significantly lower knowledge scores compared to Europe (mean difference − 0.78309, significance < 0.001), and Australia/Oceania (means difference − 0.84316, significance < 0.001). North Americans (Other) also showed significantly lower knowledge scores compared to Europe (means difference − 0.76122, significance 0.001) and Australia/Oceania (means difference − 0.84316, significance 0.001). Values from Asia, Africa, and South American should be looked at with caution because of low responses. Antarctica was excluded from these calculations because there was only one respondent.

Analysis of respondents highest level of education completed showed a Welch statistic of 13.030 and significance of 0.001 (*p* < 0.05). Post hoc showed that those who completed a Professional degree had significantly higher scores than Bachelor’s degree (means difference 0.55353, significance of 0.007), Associates degree (means difference 1.21578, significance of < 0.001), and high school (means difference 1.11273, significance < 0.001). Those with Masters Degrees were significantly higher scoring than Associate degrees (means difference of 0.80000, significance < 0.001), and high school (means difference of 0.69695, significance of 0.001). Bachelor’s degree holders had significantly higher scores compared to Associates degree (means difference of 0.66224, significance of < 0.001) and High school (means difference of 0.55920, significance of 0.003). Those values from who have no formal school or only completion of elementary school should be looked at with caution due to low frequencies.

Socioeconomic class compared to knowledge scores yielded a Welch statistic of 0.266 and a significance of 0.767 (*p* > 0.05). No further analysis was needed.

The type of social media used compared to knowledge score showed a Welch statistic of 7.175 and significance of < 0.001(*p* < 0.05). Games Howell determined that Twitter users had significantly higher scores than Facebook (means difference 0.43812, significance of 0.001) and Instagram (means difference 0.69491, significance of 0.001).

Hours spent on social media showed a Welch statistic of 2.531 and significance of 0.044 (*p* < 0.05). Post hoc testing showed significantly lower values in those who use social media for 3–4 h compared to 0–2 h (means difference 0.33869, significance of 0.018). No other means from this analysis were significant.

Whether or not a respondent had seen anything on social media about vaccines was not analyzed because there are only 2 categories and therefore the question is noncompliant with the Welch analysis. The influence of vaccine posts on social media had a Welch statistic of 145.202 with a significance of < 0.001 (*p* < 0.05). Post hoc testing revealed that those who now perceived their opinion of vaccine of being worse than previously thought had significantly lower scores compared to those who now think vaccines are better (means difference − 6.36712, significance of < 0.001), no influence/change in opinion (means difference − 5.83564, significance of < 0.001) and those who had not seen anything (means difference − 4.70483, significance of < 0.001). Those who think vaccines are better after seeing social media posts had significantly higher scores compared to worsened opinions (as mentioned before), those who were not influenced (means difference 0.53148, significance < 0.001) and those who have not seen anything (means difference 1.66229, significance < 0.001). In addition, those who have not been influenced by posts had significantly higher scores than those who have not seen any posts (means difference 1.13081, significance < 0.001).

Lastly, those trusted for immunization related information and decisions was analyzed and found a Welch statistic of 83.032 with significance of < 0.001 (*p* < 0.05). Post hoc analysis showed those who trusted Doctors the most have significantly higher scores than those who trusted the internet (means difference of 5.32139, significance of < 0.001), family (means difference 5.94306, significance < 0.001), and peers (means difference 6.31957, significance of < 0.001). Those who trusted the government the most also had significantly higher scores than internet (means difference 5.13429, significance < 0.001), family (means difference 5.75595, significance of < 0.001) and peers (means difference 6.13247, significance of < 0.001). Trusting of social media should be looked at with caution due to low frequencies.

### Beliefs

Depiction of the frequency of belief scores in the sample population can be found in Table 2, found in the supplementary document 2 labeled “Tables”. As noted in the methods, the remaining 6 questions were scored on a two point scale resulting in a belief score from 0 to 12. Scores toward 0 represent negatively skewed beliefs or belief in common myths. Scores toward 12 represent positively skewed beliefs or disbelief in common myths. Scores of 6 represent uncertainness.

The analysis of the demographic questions against the individual’s belief score was completed by Welch and then further analyzed by Games Howell. Explanations of why these tests were chosen can be found in methods. When age was compared with belief score a Welch statistical value of 2.923 and significance of 0.013 (*p* < 0.05). Post Hoc revealed 65-year-olds and older had significantly lower scores than 10–24-year-olds (mean difference − 1.37750, significance 0.014) and 24–34 year olds (mean difference − 1.19606, significance 0.047).

Gender analysis showed a Welch statistic of 0.320 with a significance value of 0.728 (*p* > 0.05). Post hoc analysis was not examined because there was no significance.

Geographical Welch testing showed a statistic value of 29.212 with a significance of < 0.001(*p* < 0.05). Due to this value being statically significant, post hoc analysis was examined. North Americans (USA) had significantly lower belief scores compared to Europe (mean difference − 1.47989, significance < 0.001), and Australia/Oceania (means difference − 1.81575, significance < 0.001). North Americans (Other) also showed significantly lower belief scores compared to Europe (means difference − 1.29021, significance < 0.001) and Australia/Oceania (means difference − 1.62607, significance < 0.001). Values from Asia, Africa, and South American should be looked at cautiously because of the low response rate. Antarctica not included in these calculations because there was only one individual who responded.

The analysis of individuals with the highest level of education completed showed a Welch statistic of 17.789 and significance of < 0.001 (*p* < 0.05). Post hoc showed that those who completed a professional degree had significantly higher scores than those with master’s degree (mean difference 0.74516, significance of 0.009), bachelor’s degree (means difference 1.10881, significance of < 0.001), associate’s degree (means difference 2.02797, significance of < 0.001), and high school (means difference 1.97009, significance < 0.001). Respondents with master’s degrees were significantly higher scoring than those with associate degrees (means difference of 1.28280, significance < 0.001), and high school (means difference of 1.22493, significance of < 0.001). Those with bachelor’s degrees had significantly higher scores compared to those with associate’s degrees (means difference of 0.91916, significance of < 0.001) and high school (means difference of 0.86128, significance of < 0.001). The values from those individuals that had no formal schooling, or only completion of elementary school, should be looked at cautiously due to low frequencies.

Socioeconomic class, compared to belief scores, resulting in a Welch statistic of 0.028 and a significance of 0.972 (*p* > 0.05). No further analysis was needed.

The type of social media users compared to belief score showed a Welch statistic of 8.011 and a significance of < 0.001(*p* < 0.05). Games Howell determined that Twitter users had significantly higher scores than Facebook (means difference 0.55094, significance of 0.001) and Instagram (means difference 0.98733, significance of < 0.001).

Hours spent on social media showed a Welch statistic of 3.162 and a significance of 0.016 (*p* < 0.05). Post hoc testing showed significantly lower values in those who used social media for 3–4 h compared to 0–2 h (means difference 0.39195, significance of 0.034). No other means from this analysis were significant.

Exposure to posts on social media about vaccinations was not analyzed because there were only two categories and therefore, noncompliant with the Welch analysis. The influence of vaccine posts on social media had a Welch statistic of 312.900 with a significance of < 0.001 (*p* < 0.05). Post hoc testing revealed that those who now perceived their opinion of vaccines of being worse than previously thought, had significantly lower scores compared to those who now think vaccines are better (means difference − 7.97280, significance of < 0.001), No influence or change in opinion (means difference − 7.27248, significance of < 0.001), and those who had not seen anything (means difference − 4.97992, significance of < 0.001). Those who thought vaccines were better after seeing social media posts had significantly higher scores compared to worsened opinions, as mentioned before, and to those who were not influenced (means difference 0.70031, significance < 0.001), and those who had not seen anything (means difference 2.99288, significance < 0.001). Also, those who had not been influenced by social media posts had significantly higher scores than those who had not seen any posts at all (means difference 2.29256, significance < 0.001).

Finally, those trusted in immunization related information and decisions were analyzed and found a Welch statistic of 150.953 with a significance of < 0.001 (*p* < 0.05). Post hoc analysis showed those who trusted doctors the most had significantly higher scores than those who trusted social media (means difference of 6.50713, significance of < 0.001), family (means difference 7.28796, significance < 0.001), and peers (means difference 7.41258, significance of < 0.001). Individuals who trusted the government the most also had significantly higher scores than social media (means difference 6.87265, significance < 0.001), family (means difference 7.65349, significance of < 0.001) and peers (means difference 7.77811, significance of < 0.001). Trusting of social media should be looked at cautiously due to its low frequencies.

## Discussion

The significance found in this study can help us understand who is being influenced by posts about vaccines on social media. It is important to note that the sample in this study was originally started in North America; hence the vast majority of respondents reported residing on this continent. Beyond that, significant values from the statistics should be examined to determine what it means for this research and for further implications.

### Mean scores

Overall this study found that respondents were very knowledgeable with a mean knowledge score of 10.4. Very few people had negatively skewed knowledge 0–4 (138 people, 5.7% of the total study population). Further investigation into those people that scored lowest would be able to show greater detail into the minds of those people and where the lack of factual knowledge is coming from or what hurdle needs to be faced.

Respondents had mainly positive beliefs about vaccines with a mean belief score of 9.68, with a standard deviation of 3.14. Few people had negatively skewed beliefs 0–4 (236 people, 9.8% of the total study population). Looking deeper into the individuals that had lower scores would be able to show greater explanation into why their beliefs about vaccines were negative, and how we might be able to change these beliefs.

### Age

In a study in 2009, 75.64% of people aged 18–64 were internet users and 74% of users aged 18–24 were social media users (Chou et al). This is important because the high social media use group from that study is now 27–31 years old and could now be making decisions about vaccines for their children.

When looking at ages, we saw that 65 years and older tend to have more negative beliefs than those individuals that are 18–24 and 25–34, with a significant difference of 0.013 (*p* < 0.05). Meaning, older people tend to believe the things they see on social media more than someone who is younger. We saw that in the previous research, older people are less likely to use social media [4], but in this study, those who did use social media scored lower. There were no significant correlations regarding age for knowledge scores.

### Gender and socioeconomic status (SES)

A comprehensive research paper by UNICEF in 2013 [36] titled “Tracking anti-vaccination sentiment in eastern European social media networks” found that females are more likely to discuss developmental disabilities, chemical, toxins and potential side effects whereas males are more likely to discuss conspiracy theories, religion and distrust of the government.

Across all genders and all SES, we saw no significant differences in knowledge or beliefs. This is significant to note because as previous literature and this one found, women are the predominant social media users. Since gender and SES have no impact on vaccine knowledge or beliefs we look deeper into other variables.

### Geography

For geography, analysis was according to continent lived on and subsequently found statistical significance. North Americans compared to all continents are significantly less knowledgeable about vaccines and had more negatively skewed beliefs. Although this research does not give a reason to why North Americans are less knowledgeable and believe more myths it gives us insight into areas that need to be studied.

There was little data available on Asia, Africa, and South America and none on Antarctica making analysis unreliable and therefore all of which should be disregarded. It is evident that Asia is showing a lower mean knowledge than other areas but without significance due to low frequency. This is paralleled when looking at beliefs. This may be explained by previous research has found that “among people who use the internet, those in developing countries often turn out to be more likely than their counterparts in advanced economies to network via platforms like Facebook and Twitter” [33]. Further investigation should determine if Asian countries do in fact have lower knowledge of vaccines and why this is. Some things to determine include whether there is a quantifiable difference between continental education, social media, use, cultural perceptions of vaccination, etc.

### Education

There is a steady incline in mean knowledge and mean belief score as education level increases. Due to low response frequencies of “no education” and “elementary education” results pertaining to these categories should be disregarded. There are significant differences between all other education levels in an increasing fashion for both knowledge and beliefs. This tells us that those with higher education are in some way or another seeking and finding valid information about vaccines. “Formal schooling adds significant value to innate ability in the form of higher-order cognitive skills crucial to decisions about health” [2]. This is important in terms of whom to focus on for future vaccine education and propaganda.

### Social media platform

A comprehensive research paper titled “Tracking Anti Vaccination Sentiment in Eastern European Social Media Networks” by UNICEF in 2013 [36] found that vaccine influencers (people or pages that speak publicly about vaccines, both positively and negatively) are most prominent on Facebook and Twitter. This study found that Twitter users are significantly more knowledgeable about vaccines than Facebook or Instagram users and also had more statistically significant positive beliefs about vaccines. Somehow, information is reaching Twitter users but not reaching other forms of social media. Upon further investigation research has showed “information sought from Facebook may be obtained socially (i.e. by asking other users), whereas the information sought on Twitter might be more cognitively based, such as academic or political information that is best gained by reading source materials, for which links are often ‘tweeted’ “[22].

### Time on social media

While the majority of people claimed to only spend 0–2 h on social media daily, those who spent more time, namely 3–4 h, were significantly more knowledgeable about vaccines and had significantly more positive beliefs about vaccines. Although not explored in this study, other studies have found that upwards of 90% of young adults use social media and this number is increasing every year and the majority of this time is spend on smartphones [37]. Possible reasons for the increased scores may be that those who spend more time on social media could be spending that time reading more deeply into conversations or information. Although it does not appear to be currently studied, there could also be an association between access to a smartphone and the ability to fact check information on the spot.

### Vaccine related posts

One of the most interesting takeaways from the data analysis is how vaccine posts have influenced opinions. Those who reported that after seeing vaccine posts they now think vaccines are worse have significantly lower knowledge and belief scores. The opposite is true for those who reported more positive opinions since seeing posts, their scores were significantly higher. The importance of this is that people are able to accurately self-report how these vaccine posts affect them. Those people who see posts about vaccines that make them think vaccines are “bad” have less knowledge or their knowledge has been changed from correct to incorrect with these posts.

A European study found that over 40% of respondents had some degree of “negative feelings about vaccine safety” [28]. These “fear” posts are typically full of myths, although not inherently known to be myths by the reader, about vaccines and as a result people became more likely to believe the myths. While it cannot be inferred why this is happening it is an interesting statistic. The presence of this significance lets health/vaccine promoters, such as government; know that the vaccine posts with valid knowledge and promotion are working. Those respondents that reported they were not influenced by social media posts, had lower belief scores compared to those who said they were positively influenced by social media posts. Those who reported they were not influenced had similar knowledge scores to those who were positively influenced. This may be due to the ability to weed through information to find facts versus fiction.

The internet is now the easiest and most accessible way to get information. A google search from the United States of America for the term “vaccination” yielded 71% anti-vaccination pages and only 29% pro vaccination pages [25]. Many of these anti-vaccinations sites claimed that vaccines contain “poisons” and were damaging to the human body [25]. “In many cases, [those who perceive vaccines as harmful] may become the only individuals who voice their opinions [32]. This allows for a greater propagation of anti-vax information rather than pro-vaccination and factual knowledge. Googling information about the MMR vaccine and autism only 51% of the search results yielded correct information [41]. Not only is it important to understand the diversity of search results but also the quality of the websites appearing. Many of “sites masquerading as official scientific sites, some web users may not question the veracity of such material” [12]. Although this study does not specifically focus on the internet, social media allows for easy sharing of not only opinions but also links to some of these “pseudo-science” websites.

### Trusted source

Lastly, the analysis of who people trust most with their vaccine related information and decisions. Literature review revealed a study that found that parents who used the internet to get their information about vaccines were more likely to think children do not benefit from vaccines [24]. It is comforting to know that the vast majority of respondent in this study do in fact rely on the information they receive from their doctor and government. While many anti-vax campaigns are grounded on the fact that authorities are misconstruing vaccine information, the idea of big pharma, it is evident that that ideal is not being taken up by social media users. Those who trust the government and doctors with their information were significantly more knowledgeable about vaccine and more positive beliefs facts than those who trust another group. While it is known that many anti-vaxxers rely on social media to disseminate their message, very few people trust social media as a source. This is very good news for doctors whose patients seek information from them and government run websites such as health departments and health organizations.

### Limitations

Some limitations faced by this research include the cross sectional nature of this study. Cross sectional studies cannot infer causation, only correlations. It also limits this survey to only surface information and not important individual details. In addition, a limitation may be that demographics were not cross analyzed. This means that while it was found that professionals had the highest knowledge score this research does not delve into the gender, location, age, etc. of these people. This same information could be analyzed in this way for further investigation. It is also important to note that this information is limited by some frequencies being too low for confidence. These areas would ideally have to be isolated and looked at again with higher number of participants. These categories were mentioned in the discussion and results stating they should be disregarded.

This study is also limited by the snowball sampling method. This limits the study by which participants decided to share, and where the majority of their friends are located, as seen by the North American predominance. While great caution was taken when constructing this survey to be nonjudgmental, some valuable information is lost in the fact that respondents were not asked their vaccine practices.

As with all online surveys, no interviewer was present. Respondents were not able to clarify answers that they felt needed explanation. Without an interviewer there is no probing into deeper answers. Information this study found could be used in the future for interviewer based research to determine a more in depth understanding of those who lacked vaccine knowledge. Another limitation of being an online survey is that there is no accountability for answers. Respondents could have clicked through the survey to complete all the questions as quickly as possible without regard for what the answer choices were.

The information portrayed in this research by the authors favors scientific fact as described by other journal articles and not personal opinions/bias. Every effort was made not to use incriminating or offensive language and statements in the creation of the survey. This core of this research was completed by the authors when they were medical students. Saint James School of Medicine ethics committee approved this research and all of its modalities. Survey platform was provided by Saint James School of Medicine.

## Conclusions

As social media continues to grow exponentially, it can be expected that anti-vaxxers will further spread their messages across these platforms. While this research does not delve into the totality and extent of the anti-vax movement on social media it does provide some insightful information. As mentioned previously, it is possible for governments and doctors to use this information to intervene and correct false information about vaccines and their safety. Given that trust in doctors resulted in significantly higher knowledge and belief scores, it is imperative that physicians create a trusting environment and relationship with their patients and guardians of pediatric patients. This also tells us that the information patients are getting from their doctors and the conversations they are having are influencing vaccine decision making in a positive way. What was not studied, and would be beneficial to investigate going forward, is whether doctors have the ability to change a patient’s perspective on vaccines once they have been exposed to negatively skewed vaccine information. It is also evident that those who trust the internet with their vaccine information are exposed to more misinformation. There may be an avenue for addressing misinformation on the internet through this trusted source and that could be via physician social media influencers. We have started to see the importance of this avenue with the prevalence of COVID-19. Social media has create an easily accessibly way to reach credible sources such as CDC and WHO, who made their presence known during the height of the pandemic through social media campaigns [7]. This appears to be especially important for direct intervention on Facebook, where the majority of social media use is, and unfortunately lower rates of knowledge and more negatively skewed beliefs. In North America, actions should be taken to combat the misinformation and myths that are reaching its population more than anywhere else in the world.

Further research is recommended to understand why some countries, age groups, social network users, etc. are not getting adequate information on vaccines. Future research would benefit from using a structured interviewer based approach to allow for expansion and clarification of answers. Further evaluation of the data collected here could yield more in-depth understanding of demographics. As mentioned before this research did not cross analyze demographics.

## Supplementary Information

**Additional file 1.** Survey. Anti- Vax research survey. This file includes the questions and multiple choice options that were used to build the research’s SurveyMonkey Survey.

**Additional file 2: Table 1.** Frequency of Knowledge Scores 0-12 with percent of total sample population. **Table 2.** Frequency of Belief scores 0-12 with percent of total sample population.

## Data Availability

Raw and descriptive data is available from openICPSR.org project ID openicpsr-120505.

## Abbreviations

Anti-Vax/ Anti- vaxxer: Anti- vaccinator / Vaccine denier
HPV vaccine: Human Papilloma Virus vaccine
MMR vaccine: Measles Mumps Rubella vaccine
UNICEF: United Nations International Children’s Emergency Fund
SPSS: Statistical package for social sciences
SES: Socioeconomic status
