# Supplementary material for: The “anti-vax” movement: a quantitative report on vaccine beliefs and knowledge across social media
Source: BMC Public Health. 2021 Nov 17;21:2106. doi: 10.1186/s12889-021-12114-8 (PMC8596085; doi:10.1186/s12889-021-12114-8)
Supplement: Supplementary file 1 — Additional file 1. Survey. Anti- Vax research survey. This file includes the questions and multiple choice options that were used to build the research’s SurveyMonkey Survey. [file 12889_2021_12114_MOESM1_ESM.docx]

| Anti-Vax Research Survey | |
| --- | --- |
| Questions | Answer Choices |
| This is not a test, there are no correct answers. This survey seeks to determine attitudes toward and knowledge about vaccination in social media users. Participation in this survey is completely voluntary. All answers will be anonymous. By participating in this study you agree that all of your answers are honest and true. This study includes some personal demographic questions which must be answered for completion of this survey. You are not obligated to complete the survey and may stop at any time. Those who choose not to complete the entire survey will be excluded from research without consequence. Permission is needed to use research data from this survey toward the researchers publication on this topic. You must be 18 years or older to complete this study. Data collection is managed by SurveyMonkey. Please review their Privacy Policy to better understand how your information is collected, secured, and managed. By answering “ I Agree” below you consent to the information in the survey being used for research analytics, you confirm that you are 18 years if age or older, and that you agree to the terms as described above. | - I agree to consent, data use, and terms listed above. |
| What is your age? | - 18-24 |
|  | - 25-34 |
|  | - 35-44 |
|  | - 45-54 |
|  | - 55-64 |
|  | - 65+ |
|  |  |
| What is your gender? | - Female |
|  | - Male |
|  | - Other / Prefer not to answer |
|  |  |
| Which continent do you live on? | - North America, United States |
|  | - North America, Other |
|  | - South America |
|  | - Europe |
|  | - Africa |
|  | - Asia |
|  | - Australia/ Oceania |
|  |  |
| What is the highest level of education you have completed? | - No formal schooling |
|  | - Elementary school (grade level 1-8) |
|  | - High school (grade level 9-12/13) |
|  | - Associates Degree (2 year college/university degree) |
|  | - Bachelor Degree (4 year college/university degree) |
|  | - Master's Degree |
|  | - Professional/Doctoral Degree (PhD, MD, DC etc..) |
|  |  |
| What socioeconomic class would you consider yourself/ your family? | - Lower Class |
|  | - Middle Class |
|  | - Upper Class |
|  |  |
| Which form of social media do you use most? | - Other (please specify) |
|  | - Facebook |
|  | - Twitter |
|  | - Instagram |
|  |  |
| In a typical day, how many hours do you spend on social media? | - 0--2 |
|  | - 3--4 |
|  | - 5--6 |
|  | - 7--8 |
|  | - 9+ |
|  |  |
| Have you ever seen any posts about vaccines on social media? | - Yes |
|  | - No |
|  |  |
| Information about vaccines seen on social media has: | - Influenced my opinion on vaccines (vaccines are worse than I thought previously) |
|  | - Influenced my opinion on vaccines (vaccines are better than I thought previously) |
|  | - NOT influenced my opinion on vaccines |
|  | - I have not seen anything about vaccines on social media |
|  |  |
| Who do you trust most with immunization related information/ decisions? | - Doctors |
|  | - Internet |
|  | - Family |
|  | - Peers/Friends |
|  | - Social media |
|  | - The government |

| The MMR (Measles Mumps and Rubella) vaccine causes autism |  |
| --- | --- |
|  | - I believe this is true |
|  | - I believe this is NOT true |
|  | - I am unsure |
| Most diseases are so rare that they no longer require vaccination. |  |
|  | - I believe this is true |
|  | - I believe this is NOT true |
|  | - I am unsure |
| Parents, rather than the government, should decide if a child gets vaccinated. |  |
|  | - I believe this is true |
|  | - I believe this is NOT true |
|  | - I am unsure |
| The mercury and aluminum used in vaccines is dangerous and can cause serious neurological problems. |  |
|  | - I believe this is true |
|  | - I believe this is NOT true |
|  | - I am unsure |
| A child's immune system cannot handle multiple vaccinations at one time. |  |
|  | - I believe this is true |
|  | - I believe this is NOT true |
|  | - I am unsure |
| The CDC (Center for Disease Control) and/or Drug companies are not trustworthy. |  |
|  | - I believe this is true |
|  | - I believe this is NOT true |
|  | - I am unsure |
| It is important to complete all vaccines in the recommended time of a series. |  |
|  | - I believe this is true |
|  | - I believe this is NOT true |
|  | - I am unsure |
| The risk of death from receiving a vaccine is estimated to be less than 1 in a million people. |  |
|  | - I believe this is true |
|  | - I believe this is NOT true |
|  | - I am unsure |
| The measles vaccine has led to a 99% decrease in cases of measles. |  |
|  | - I believe this is true |
|  | - I believe this is NOT true |
|  | - I am unsure |
| An additional 1.5 million deaths could be avoided if global immunization rates improve. |  |
|  | - I believe this is true |
|  | - I believe this is NOT true |
|  | - I am unsure |
| Data collected from the Vaccine Adverse Event Reporting System (VAERS) is available to the public and accessible online. |  |
|  | - I believe this is true |
|  | - I believe this is NOT true |
|  | - I am unsure |
| Immunization of large portions of the population is needed in order to protect immunocompromised individuals (people who cannot receive vaccines). |  |
|  | - I believe this is true |
|  | - I believe this is NOT true |
|  | - I am unsure |
