# Supplementary material for: The “anti-vax” movement: a quantitative report on vaccine beliefs and knowledge across social media
Source: BMC Public Health. 2021 Nov 17;21:2106. doi: 10.1186/s12889-021-12114-8 (PMC8596085; doi:10.1186/s12889-021-12114-8)
Supplement: Supplementary file 2 — Additional file 2: Table 1. Frequency of Knowledge Scores 0-12 with percent of total sample population. Table 2. Frequency of Belief scores 0-12 with percent of total sample population. [file 12889_2021_12114_MOESM2_ESM.docx]

**Table 1**

| **Knowledge Score** | **Frequency** | **Percent** |
| --- | --- | --- |
| **0** | 18 | 0.7 |
| **1** | 15 | 0.6 |
| **2** | 67 | 2.8 |
| **3** | 17 | 0.7 |
| **4** | 21 | 0.9 |
| **5** | 25 | 1.0 |
| **6** | 35 | 1.4 |
| **7** | 42 | 1.7 |
| **8** | 84 | 3.5 |
| **9** | 141 | 5.8 |
| **10** | 318 | 13.2 |
| **11** | 499 | 20.6 |
| **12** | 1135 | 47.0 |
| **Total** | 2417 | 100 |

Table 1: Frequency of Knowledge Scores 0-12 with percent of total sample population.

**Table 2**

| **Belief Score** | **Frequency** | **Percent** |
| --- | --- | --- |
| **0** | 59 | 2.4 |
| **1** | 39 | 1.6 |
| **2** | 46 | 1.9 |
| **3** | 28 | 1.2 |
| **4** | 64 | 2.6 |
| **5** | 56 | 2.3 |
| **6** | 79 | 3.3 |
| **7** | 76 | 3.1 |
| **8** | 126 | 5.2 |
| **9** | 160 | 6.6 |
| **10** | 335 | 13.9 |
| **11** | 354 | 14.6 |
| **12** | 995 | 41.2 |
| **Total** | 2417 | 100 |

Table 2: Frequency of Belief scores 0-12 with percent of total sample population.
